# Factors Associated with and Predictive Model for Resilience in Family Caregivers of Care-Dependent Adults

**DOI:** 10.3390/nursrep14040253

**Published:** 2024-11-12

**Authors:** Belen Gutierrez-Baena, Ester Gilart, Carmen Romero-Grimaldi

**Affiliations:** 1 Nursing Faculty Salus Infirmorum, University of Cadiz, 11001 Cadiz, Spain; belen.gutierrezbaena@ca.uca.es; 2 Hospital “Viamed Bahía de Cadiz”, 11130 Cadiz, Spain; 3 Department of Nursing and Physiotherapy, University of Cadiz, 11009 Cadiz, Spain; ester.gilart@uca.es; 4 Centro de Investigación Biomédica en Red de Salud Mental (CIBERSAM), Instituto de Salud Carlos III, 28029 Madrid, Spain

**Keywords:** caregivers, resilience psychological, anxiety, caregiver burden, risk factors, nursing

## Abstract

**Background:** Caring for dependent people is an intense task that leads family caregivers to suffer physical or mental pathologies. Resilience is a protective factor that makes an individual more resistant to adverse events. Some characteristics of the caregiver or the care provided predispose the caregiver to be less resilient. Knowing these characteristics will allow us to detect vulnerable caregivers. **Aim:** The aim of this study was to explore the factors associated with caregiver resilience and establish a predictive model, including the relationship between preparedness, burden, resilience, and anxiety. **Materials and Methods:** The study design was descriptive, observational, and cross-sectional, with purposive sampling. The sample included 172 family caregivers of care-dependent patients in Spain. Questionnaires were administered to assess caregiver resilience, anxiety, burden, and the preparedness of care-dependent patients. Correlation, univariate, and multiple linear regression analyses were performed to identify the factors associated with resilience. **Results:** We found that there is a correlation between resilience, preparation, and burden. Factors related to resilience include age, the caregiver’s health status, kinship, childcare, and state aid granted. Caregiver preparedness is the factor that most predicts caregiver resilience in our model. Caregivers with high levels of resilience also demonstrated high levels of preparedness and experienced less burden than those with low levels of resilience. **Conclusions:** These findings emphasize the relevance of improving the preparation of family caregivers to increase their resilience and, at the same time, the quality of care provided.

## 1. Introduction

Caregivers provide continuous care for the physical, mental, and emotional well-being of people. Non-professional caregivers—caregivers who are unpaid—have expressed that they are not prepared for the irreplaceable role that can sometimes suddenly occur [1]. In addition, many family caregivers have other obligations such as their own job, childcare, home maintenance, health, or additional worries. In Spain, to alleviate the family overload that these situations entail, there are social aids that benefit patients with a high degree of dependency, which is regulated under the so-called Dependency Law. In 2006, the Spanish Dependency Law established new rights for people in situations of dependency. The Dependency Law provides access to many services and benefits that are funded partially or totally by the state for dependent people of all ages. There are three levels of dependency, which are medically assessed using a point system [2]. Indeed, this system is helpful for many caregivers and alleviates part of their responsibility. However, it is important to note that many caregivers do not request it, others are not eligible to receive it, and many others give up due to the bureaucratic procedures involved.

When the demands of caregiving exceed the caregiver’s ability to provide, it directly affects their quality of life [3], carrying a set of negative consequences that can drastically affect the caregiver and produce a burden [4]. This concept is widely attested to having a negative impact on the well-being of family caregivers [5], defined as the perception of deterioration in emotional or physical health, social life, and economic status while caring for their family members [6]. There is a direct relationship between caregiver burden, anxiety, and depression [7]. Specifically, as caregiver burden increases, so do levels of anxiety and depression [8,9,10]. In the opposite direction, it has been described that caregivers who receive some type of help can reduce their burden [11,12]. The level of preparedness has the same effect, with the best-prepared caregivers being those who experience less burden [13] and have a better health status [14]. Therefore, taking into account the negative consequences of caregiver exhaustion, we must try by all possible means to avoid or prevent it.

Although we know that some caregivers do not experience high levels of burden and also have much better coping skills, they are the ones who have greater resilience [15]. The concept of resilience has evolved. Rutter defined it as the phenomenon by which individuals achieve relatively good results despite being exposed to adverse experiences [16]. However, for other authors, resilience is not only a mere resistance to adversity but also a person’s ability to adapt to change successfully, sustain the negative impact of stressors, avoid the occurrence of significant dysfunction [17], and grow in difficult contexts [18]. Understanding this construct and its associated psychological factors can promote a more effective adaptation to stress and reduce the potential threats of these events. Resilient caregivers exhibit determination, flexibility, positive thinking, self-efficacy, resourcefulness, social support, and spirituality [19]. In this study, we have adopted the theoretical approach of Connor and Davidson [20] to address resilience and used the measurement instrument they proposed (Connor–Davidson Resilience Scale). These authors conceive resilience as a multidimensional characteristic that integrates personal competence, tenacity, trust in one’s institution, tolerance of adversity, positive acceptance of change, the establishment of secure relationships, control, and spirituality.

Since resilience facilitates adaptation to difficult situations, a psychological aspect of personality is important in the caregiver’s life. Individuals with high resilience can adapt psychologically and physically, whereas caregivers with low resilience are at risk of poor psychosocial outcomes [21]. According to this, in caregivers of patients with Alzheimer’s, resilience appears as a protective factor against the development of psychological symptoms of a depressive or anxious type [22]. In the same sense, other studies have shown that resilience enhances caregiving readiness [23], good health status, less depressive comorbidity, and good social support from caregivers [21,24,25]. With respect to the patient, caregiver resilience is also beneficial, since it has been determined that high caregiver resilience prevents patient relapses and contributes to their recovery [26]. Although studies indicate that improving resilience is beneficial for caregivers for various reasons, there are not many interventions in this regard, possibly because of the difficulty involved in these proposals.

There are some studies in which a significant relationship between caregiver preparation and resilience has been described [13,23,27,28,29]. We know social support, how stressors are evaluated, and the coping style used are characteristics that promote resilience in caregivers [30,31,32]; we believe that caregiver preparation could be included among those factors that promote this ability to cope with problems. In our attempt to explain and delve deeper into the construct that explains caregiver readiness, the factor that decisively predicted it more than many other factors was resilience [13]. Therefore, we think that in the same way that resilience determines the level of preparation, the level of preparation will also predict the level of resilience. A sufficiently prepared caregiver will have the basic tools and skills to deal with the many situations and daily challenges that continuous care entails.

The aim of the present study is to advance our knowledge of the factors that influence certain caregivers to successfully adapt to the difficulties of daily caregiving. This will contribute to the development of a more integrated and operational model of resilience and care than the one proposed by Gaugler et al. [33], which is the only one developed to date for this purpose. Specifically, we propose studying the possible relationship between resilience and other intrinsic and extrinsic factors to caregivers and determine whether there is a correlation between our study variable and caregiver preparedness and burden. Knowledge of the relationship between these variables will allow us to determine the characteristics of caregivers with low resilience. This will allow us to direct much more effective interventions toward them.

## 2. Methods

### 2.1. Study Design and Procedure

A correlational, cross-sectional, and descriptive design study was conducted in Cadiz in southern Spain. A non-probabilistic convenience sample was used. The sample consisted of 172 primary caregivers selected from a private hospital and daycare centers. At a minimum, participants had to care for their family members for at least two hours a day and declare themselves the primary caregiver of a care-dependent person. Caregivers who lived with the dependent person and those who did not were included. The inclusion criteria were over 18 years of age, Spanish nationality, no cognitive impairment, written consent, not being a healthcare professional, and providing care to a family member with moderate, severe, or total dependency according to the Barthel index (BI) [34]. The BI is one of the most widely used tools to evaluate an individual’s ability to perform daily activities. Each task was assigned a score of 0, 5, 10, or 15 points. The sum of the scores determines the level of dependency, which varies from 0 to 100.

### 2.2. Data Collection

Data were collected between October 2018 and June 2019 by two researchers. Four instruments were used, and the sociodemographic data of the caregivers were collected using a questionnaire. The data were collected on paper forms in collaborating centers. The caregivers recruited in the hospital completed the surveys during their stay, whereas the family caregivers recruited from daytime nursing centers completed them at home.

Demographic data included age, sex, marital status, and level of studies, among others. Variables related to care included the time dedicated to the care, whether a dependent person and their caregiver live in the same home, and if the Dependency Law was granted, among other variables.

Resilience was measured using the 10-item Connor–Davidson Resilience Scale (CD-RISC10), which showed supportive fit indices during a confirmatory factor analysis (CFA, e.g., comparative fit index [CFI], 0.94) and reliability (Cronbach’s α = 0.89). Each item was rated on a five-point Likert-type scale ranging from “never” (0) to “almost always” (4), with a total score from 0 to 40. Higher scores indicated greater levels of resilience. For this study, we used an abbreviated Spanish version of the scale [35].

Preparedness was measured using the Caregiver Preparedness Scale (CPS), which was originally developed in the United States and is used to assess caregivers’ readiness [36]. The scale includes eight items, each rated on a five-point Likert-type scale ranging from “not at all prepared” (0) to “very well-prepared” (4). A total score from 0 to 32 was calculated by adding the responses for all items, with a higher score indicating a greater level of preparedness. For this study, the Spanish version of the CPS (SV-CPS) was used, which demonstrated supportive fit indices in a confirmatory factor analysis (CFA, e.g., CFI, 0.92) and reliability (Cronbach’s α = 0.89) among family caregivers [37].

Anxiety was measured using the State–Trait Anxiety Inventory (STAI), an instrument for measuring anxiety that consists of 40 items divided into two scales of 20 items. Each item was scored between 0 (almost never) and 3 (almost always). The total score ranged from 0 to 120. The higher the participant’s score, the higher their anxiety level. This instrument demonstrated good reliability in its original English and Spanish versions (Cronbach’s α ≥ 0.90) used for this study [38].

Caregiver burden was measured using the Zarit Burden Interview (ZBI), which measures a caregiver’s burden using 22 items and includes a five-point Likert-type scale, with each item scored between 0 (never) and 4 (usually). The total score, calculated by adding all the responses, ranges from 0 to 88, with a higher score indicating a greater level of burden. The ZBI is a widely used and validated test for various populations with a good index of validity (CFA, e.g., CFI, 0.96) and reliability (Cronbach’s α ≥ 0.91) [39]. The Spanish version was used in this study [40].

### 2.3. Sample Description

The sociodemographic characteristics of the sample are shown in Table 1 and determine the caregiver profile. The age of the respondents ranged from under 45 years (11%) to over 75 years (9%), with the most prevalent age range being 55–64 years (40%). The majority of participants were female (79%); married (75%); and with primary (33%), secondary (38%), or university (19%) education. Some were unemployed (12%), others were working (37%), retired (15%), or stay-at-home parents (36%). More than half of the sampled persons were daughters and sons in the care of their parents (52%), while only a few were fathers or mothers in the care of their children or spouses (17% and 16%, respectively). Their income was less than EUR 1000 or EUR 500 per month for almost half of the participants (46%). Caregivers reported being in good or very good health (55%), and others considered their health to be fair (41%) or poor (4%). Many caregivers (63%) lived in the same household as the dependent.

Of the people receiving care, 67% were female. The patients had a mean BI score of 35 (10–60). Only 22% presented a moderate or mild level of dependency, and the majority had a severe or total dependency index (78%); therefore, they needed continuous attention from the caregiver. However, the number of hospitalizations was low (≤2 in 86% of the cases) during the past year.

The characteristics related to care are presented in Table 2. Of the recruited caregivers, almost half provided care for more than 14 h a day, 44% have been caring for more than 6 to more than 10 years, and 35% have been caregivers for between 3 and 5 years. Therefore, care is a relevantly extensive and continuous activity in our sample.

In addition to caring for the dependent person, some caregivers also had dependent children (29%). Surprisingly, more than half of the sample (59%) did not receive any professional help to provide care, while some benefited from the Dependency Law, either in the form of economic benefits (16%) or help at home (24%).

### 2.4. Data Analysis

Data analysis was performed using SPSS version 21.0 statistical software. The normality of the sample distribution was analyzed using the Kolmogorov–Smirnov Z-test [41]. Nonparametric tests were used for variables that did not fit the normal distribution, and parametric tests were used for variables that fit the normal distribution. The accepted degree of significance was *p* < 0.05. The sociodemographic characteristics of the sample were analyzed using frequency analysis. Descriptive statistics (mean/median and standard deviation/interquartile range) were used to analyze the participants’ results. The relationship between the study variables was obtained by calculating bivariate correlations using Spearman’s rho coefficient. The relationship between caregiver and caregiving situation characteristics and resilience was analyzed using the Mann–Whitney U and Kruskal–Wallis H tests. Post hoc tests were conducted.

A multiple linear regression analysis was performed to explore the factors related to caregiver resilience and identify the proportion of variance explained by these variables. The factors that were significant in the univariate and correlation analyses were entered into the model as the independent variables for analysis, with resilience as the dependent variable. First, all the significant variables were entered into the model simultaneously (forced entry method). This full model was followed by stepwise backward elimination to determine whether each variable remained significant after excluding non-significant covariates. The assumptions for multiple regressions were checked using a residual analysis. The assumptions of normality, linearity, and homoscedasticity were generally met. No multicollinearity problems were detected across the independent variables; the mean level of the variance inflation factor (VIF) was within the allowed limits [42]. To consider the model valid, it must explain at least 30% of the variance; that is, the adjusted coefficient of determination must be greater than 0.300 [43].

### 2.5. Ethical Considerations

This study was approved by the relevant clinical research ethics committee. All the participants were informed about the purpose of the study and the confidentiality of the data collected and signed their informed consent forms before participating.

## 3. Results

### 3.1. Descriptive Analysis and Relationship Between Variables

The mean scores of the caregivers on resilience, preparedness, anxiety, and burden scales are shown in Table 3.

Caregiver resilience was directly related to caregiver preparedness (*r* = 0.533; *p* < 0.001) and inversely related to caregiver burden (*r* = −0.280; *p* < 0.001). We found no statistically significant associations between resilience and anxiety. Additionally, we identified a significant relationship between burden and anxiety (*r* = 0.320; *p* < 0.001) and preparedness (*r* = −0.296; *p* < 0.001). Also, we found a significant association between caregiver burden and the dependency level of the patient (*r* = −0.150; *p* < 0.05).

### 3.2. Related Factors with Resilience

In the univariate study with sociodemographic factors, we found that resilience was statistically associated with 5 of the 16 factors analyzed (Table 4). Among these were the age of the caregiver, kinship, health of the caregiver (*p* < 0.001), childcare, and the Dependency Law (*p* < 0.05), while it was not significantly associated with sex, employment status, educational level, and income of the caregiver, living with the dependent person, or having care support (family or professional). We found higher levels of resilience among young caregivers (<45 years), those with dependent children, and those with better health status. The sons-in-laws presented higher resilience values. Caregivers of relatives who did not enjoy the Dependency Law had higher levels of resilience. Table 4 shows the differences between the groups and highlights the group with the greatest resilience.

### 3.3. Predictive Model for Resilience

A multiple regression analysis was used to examine the factors related to caregiver resilience using caregiver resilience as the dependent variable and the significant factors in the univariate and Spearman’s rho correlation analysis as independent variables (Table 5). Higher caregiver resilience was significantly and strongly associated with higher preparedness (β = 0.511; *p* < 0.001) and moderately associated with good health status (β = 0.203; *p* < 0.05). Thirty-four percent of the variance in caregiver resilience was explained by the abovementioned factors (Table 6).

Preparedness and health conditions explained 51% and 20% of the variance in caregiver resilience, respectively. Specifically, the greater the level of preparedness and better the health status, the greater the resilience of the caregiver. Following the residual analysis, the proposed regression model complied with the assumptions of autocorrelation (Durbin Watson = 2.059), collinearity (VIF = 1.041 and 1.041), linearity, normality, and homoscedasticity.

## 4. Discussion

We addressed the construct of resilience by looking for correlations with other variables, such as preparation and burden, and describing the internal or external factors of the family caregivers to predict the groups that require more attention in clinical practice.

The female gender is inherent to the role of caring. In our sample and other European populations, female caregivers [3,44] were the main caregivers for family members (79% women compared to 21% men), although they did not show different levels of resilience. The profile of the majority of caregivers is a middle-aged person (the majority between 45 and 64 years), married (75%), and with a basic level of education. In most cases, the primary caregiver lived (63% vs. 37%) with the dependent person in the same home and spent many hours a day caring for them (45% spent more than 14 h). Regarding kinship, there are a variety of caregivers, although the majority are sons or daughters caring for parents and parents caring for offspring or spouses. Specifically, spousal caregivers are normally older adults who often have pathologies and deteriorating health, making care provision difficult [45,46]. The resilience capacity of our sample was similar to that of studies in very culturally diverse populations [47], and they were not different according to their gender, level of education, or length of experience. Still, there were differences with respect to other characteristics that we will comment on in this discussion. Likewise, the caregivers had medium levels of burden, preparation, and anxiety, and we have established a significant relationship between the level of resilience with the preparation and burden of the caregivers. The findings support other results that have established a direct relationship between preparation and the ability to face adverse situations [13,23,27], while burden is negatively correlated with resilience [48]. Therefore, preparedness enhances resilience, allowing caregivers to face care difficulties with resolution skills and abilities. On the other hand, caregivers with low resilience will have a lower coping capacity that will cause suffering and burden in the face of difficulties.

Contrary to expectations and as other studies have shown [49,50,51], we have not found a correlation between anxiety and resilience. This could be due to the small sample size.

Additionally, and as described by other studies, a correlation between caregiver burden and the patient’s degree of dependency has been reported [52,53]. Our results confirm this hypothesis, since our caregivers experience a greater burden when the score on the BI is lower (greater dependency). We know that highly dependent patients need help with all basic activities of daily living; therefore, the caregiver will not be able to leave the patient alone, meaning that they have to give up maintaining their self-care. This also occurs in caregivers of patients with psychiatric pathologies [54] and dementias such as Alzheimer’s [55] and cancer [56]. In conclusion, it is essential for caregivers to take time for self-care and respite, especially for caregivers of highly dependent patients who are growing in population due to the increase in life expectancy.

Certain characteristics of the caregiver or care situation determine resilience. Health status is one of the factors that has obtained the greatest significance not only with resilience (own results) but also with preparedness [13] and burden ([57] and unpublished data). Furthermore, in our predictive model of resilience, the caregiver’s health remains a decisive factor after multivariate analysis, confirming its relevance. When the health of the caregiver deteriorates, many other factors are highly affected, since the caregiver will not be able to provide care and the feeling of burden will be greater, especially if we do not have other care options. In most cases, these situations lead to the institutionalization of the patient. These results have been supported by previous studies that concluded that poor physical and mental health status caused a decrease in resilience [58]. Health status and age are commonly related variables. It is very likely that older people who become caregivers, despite their unconditional dedication in most cases, will experience problems carrying out their role, either because their health is weak or because they have low levels of resilience or preparedness. In more severe situations, deterioration in mental or physical health leads to problems such as depression or chronic pain [59,60], among others. In summary, we find that elderly caregivers and those with poorer health are more likely to face challenges in providing care and may also suffer from other concomitant pathologies that we must prevent.

Like other studies, our results show that caregivers who, in addition to taking care of a dependent person, take care of minor children are significantly more resilient [61]. This could be because they are younger caregivers (65% are under 54 years of age compared to 35% who are over 55 years of age) and therefore have fewer chronic pathologies associated with age.

Paradoxically, we found that caregivers who received aid granted through the Dependency Law (Law 39/2006 in Spain) had a slightly lower level of resilience. These state aids include economic benefits or services and have an arduous bureaucratic process and more than a year of waiting. We might expect, as other authors have reported [2,48,62], that caregivers who have this social support would show greater resilience [63,64] and have a higher quality of life [2]. Analyzing our results, we have determined that these caregivers provide care to patients with a higher level of dependency. This greater burden could explain the decrease in their resilience levels.

There are models that include resilience predictors. Leung’s study determined that psychological distress was a predictor [65]; however, Anderson’s study determined that predictors of resilience are hope, mental health, and, as found in our study, health status [66]. There are many models relating to resilience in caregivers, but few have included caregiver preparedness as an independent variable, and they have also been carried out in populations that are very different from ours. Caregiver preparedness is the most powerful predictor of resilience, as we have obtained in our model. This factor explains 51,1% of the variance, while in the model by Zhao et al. [67], preparedness explained 59.5% of the resilience of caregivers of patients with brain damage, and lower percentages (27%) were obtained in caregivers of patients with cancer [28]. We are convinced that interventions aimed at training caregivers will directly improve resilience and the ability to cope with problems. Further studies are needed to explore new factors associated with caregiver resilience. Future research should explore this line to construct a theoretical and empirical model on resilience in caregivers, which includes other variables and is applicable to other populations.

### 4.1. Limitations

This study has limitations. First, we recruited a convenience sample that included only caregivers from a single region in Spain, limiting the extrapolation of our conclusions. Second, this study was cross-sectional; thus, a temporal relationship between the variables was not established. Therefore, validating the model with covariates observed over time is recommended. Third, we did not collect the “age” continuous variable. Considering the above limitations, the generalizability of our results may be limited. Future projects could explore other factors that have yet to be considered, such as the influence of spirituality, social support, and other sociodemographic variables and variables of care.

### 4.2. Implications for Practice and Research

We now know more about the factors associated with family caregiver resilience and have a predictive model that determines which are the most important determinants, with preparation being the one that most influences the resilience of caregivers. The results of this study have identified vulnerable groups (elderly people, those in poor health, or those with low levels of preparedness). Therefore, it is possible to predict and identify those caregivers who are more likely to have difficulties in performing their role and require additional care. Multidisciplinary teams have provided evidence to design more specific interventions to improve caregiver resilience. Interventions that include training for caregivers will directly improve the ability to cope with the difficulties of care.

## 5. Conclusions

More resilient caregivers feel more prepared and experience less burden. Among the factors that determine resilience in family caregivers, the preparedness and health status of the caregiver are decisive.

The most vulnerable group is those who are older, who are in poorer health and who are also poorly prepared. This group will experience the most burden, which could be partly explained by their low levels of resilience. They will therefore need greater coverage and vigilance in caregiver support programs.

## Figures and Tables

**Table 1:** Sociodemographic characteristics of family caregivers.

	*n* (%)		*n* (%)
Sex		Educational level	
Men	36 (21)	Without studies	17 (10)
Women	136 (79)	Primary studies	56 (33)
Age (years)		Secondary studies	66 (38)
<45	19 (11)	University studies	33 (19)
From 45 to 54	44 (26)	Employment situation	
From 55 to 64	69 (40)	Employee	64 (37)
From 65 to 75	24 (14)	Stay-at-home parents	62 (36)
>75	16 (9)	Retired	26 (15)
Kinship		Unemployed	20 (12)
Son or daughter	90 (52)	Rent (EUR)	
Parents	30 (17)	<501	14 (8)
Spouse	28 (16)	Between 501 and 1000	65 (38)
Brother or sister	10 (6)	>1000	93 (54)
Daughter-in-law or son-in-law	8 (5)	Health condition	
Other	6 (3)	Good or very good	94 (55)
Marital status		Regular	71 (41)
Married	129 (75)	Bad or very bad	7 (4)
Single	26 (15)	Living together	
Widower	9 (5)	Yes	109 (63)
Divorced	4 (2)	No	63 (37)
Part of a couple	4 (2)		

The data express the percentage of the total.

**Table 2:** Other variables analyzed in caregivers.

	*n* (%)		*n* (%)
Hours per day dedicated to caregiving		Professional help	
From 2 to 8	52 (30)	Yes	70 (41)
From 9 to 13	42 (24)	No	102 (59)
≥14	78 (45)	Familial help	
Years spent caregiving		Yes	112 (65)
From 0 to 2	35 (20)	No	60 (35)
From 3 to 5	61 (35)	Dependency Law	
From 6 to 10	33 (19)	Granted	68 (40)
>10	43 (25)	Financial assistance	27 (16)
Childcare		Home assistance	41 (24)
Yes	49 (29)	Not granted/not requested	104 (60)
No	123 (72)		

The data express the percentage of the total.

**Table 3:** Mean scores of the caregivers on resilience, preparedness, anxiety, and burden scales.

Scales	Mean/Median	SD/IQR	Range
CD-RISC10	28	23–32	0–40
SV-CPS	21	17–24	0–32
STAI	51	45–58	0–120
ZBI	34.1	14.8	0–88

CD-RISC10, Connor–Davidson Resilience Scale 10-item; SV-CPS, Spanish Version Caregiver Preparedness Scale; STAI, State–Trait Anxiety Inventory; ZBI, Zarit Burden Interview; SD, standard deviation; IQR, interquartile range.

**Table 4:** Univariate analysis of resilience and post hoc analysis.

	Subgroup	Mean (SD)	Statistical
Age (H) ^b^	<45	30.11 (8.81)	21.968 ***
	45–54	28.25 (6.57)	
	55–64	26.71 (6.43)	
	65–75	29.08 (5.78)	
	>75	17.44 (9.29)	
Post hoc	>75 vs. 55–64 years		43.512 *
	>75 vs. 45–54 years		56.494 ***
	>75 vs. 65–75 years		60.312 **
	>75 vs. **<45 years**		70.062 ***
Kinship (H) ^b^	Mother/father	30.23 (6.65)	28.445 ***
	Son/daughter	26.38 (6.61)	
	Brother/sister	31.10 (4.33)	
	Spouse	21.57 (9.20)	
	Daughter-in-law/son-in-law	33.75 (5.42)	
	Other	28.17 (8.93)	
Post hoc	Spouse vs. mother/father		51.469 ***
	Spouse vs. brother/sister		59.136 *
	Spouse vs. **daughter-in-law/son-in-law**		−74.473 **
Health condition (H) ^b^	Good	28.86 (6.30)	14.716 ***
	Regular	25.44 (7.74)	
	Bad	16.57 (11.56)	
Post hoc	Bad vs. **good**		60.742 **
	Regular vs. **good**		21.419 *
Childcare (U) ^a^	Yes	29.14 (6.72)	
	No	25.98 (7.78)	
Post hoc	**Yes** vs. no		2.248 *
Dependency Law (U) ^a^	Granted	25.54 (7.57)	
	Not granted/ not requested	27.87 (7.56)	
Post hoc	Yes vs. **no**		2.853 *

Significant differences between groups are shown, and the group most favoring resilience is highlighted. ^a^ Mann–Whitney U test. ^b^ Kruskal–Wallis H test. * *p* < 0.05; ** *p* < 0.01; *** *p* < 0.001. SD, standard deviation.

**Table 5:** Factors related to caregiver resilience in the multiple regression analysis.

Variables	B	β	*t*	*p* Value
**Preparedness**	0.562	0.474	7.201	0.000 ***
Burden	−0.062	−0.121	−1.814	0.071
**Caregiver health**	2.153	0.160	2.407	0.017 *
Dependency Law	0.139	0.009	0.143	0.887
Childcare	−1.613	−0.096	−1.508	0.134
Relationship	−0.060	−0.010	−0.158	0.875

* *p* < 0.05. *** *p* < 0.001. R = 0.605; R^2^ = 0.366; F = 15.776; *p* < 0.001. β, standardized beta coefficient; R, multiple correlation coefficient; R^2^, adjusted coefficient of determination; F, Fisher’s F test.

**Table 6:** Final multiple linear regression analysis model of variables related to family caregivers’ resilience.

Independent Variables	B	Standard Error	β	*t*	*p* Value	95% CI	VIF
**Constant**	7.855	2.424		3.240	0.001	3.069; 12.641	
**Preparedness**	0.606	0.076	0.511	8.013	0.000	0.457; 0.756	1.041
**Caregiver health**	2.727	0.856	0.203	3.186	0.002	1.037; 4.417	1.041

Model statistics R = 0.586; R^2^ = 0.344; adjusted R^2^ = 0.336; *p* < 0.001; F = 44.025. B, linear coefficient; β, standardized beta coefficient; CI, confidence interval; VIF, variance inflation factor; F, Fisher’s F test; R, multiple correlation coefficient; R^2^, adjusted coefficient of determination.

## Data Availability

The dataset used for this study is available upon reasonable request from the authors.

## Public Involvement Statement

No public involvement in any aspect of this research.

## Guidelines and Standards Statement

This manuscript was drafted against the STROBE (Strengthening the reporting of observational studies in epidemiology) for cross-sectional studies. A complete list of reporting guidelines can be accessed via the equator network: https://www.equator-network.org/.
